# Improving health care quality for racial/ethnic minorities: a systematic review of the best evidence regarding provider and organization interventions

**DOI:** 10.1186/1471-2458-6-104

**Published:** 2006-04-24

**Authors:** Mary Catherine Beach, Tiffany L Gary, Eboni G Price, Karen Robinson, Aysegul Gozu, Ana Palacio, Carole Smarth, Mollie Jenckes, Carolyn Feuerstein, Eric B Bass, Neil R Powe, Lisa A Cooper

**Affiliations:** 1 Welch Center for Prevention, Epidemiology, and Clinical Research, Johns Hopkins University, Baltimore, Maryland, USA; 2 Division of General Internal Medicine, Department of Medicine, Johns Hopkins University School of Medicine, Baltimore, Maryland, USA; 3 Department of Health Policy and Management, Johns Hopkins Bloomberg School of Public Health, Baltimore, Maryland, USA; 4 Phoebe R. Berman Bioethics Institute, Johns Hopkins University, Baltimore, Maryland, USA; 5 Department of Epidemiology, Johns Hopkins Bloomberg School of Public Health, Baltimore, Maryland, USA; 6 Robert Wood Johnson Clinical Scholars Program, Johns Hopkins University, USA

## Abstract

**Background:**

Despite awareness of inequities in health care quality, little is known about strategies that could improve the quality of healthcare for ethnic minority populations. We conducted a systematic literature review and analysis to synthesize the findings of controlled studies evaluating interventions targeted at health care providers to improve health care quality or reduce disparities in care for racial/ethnic minorities.

**Methods:**

We performed electronic and hand searches from 1980 through June 2003 to identify randomized controlled trials or concurrent controlled trials. Reviewers abstracted data from studies to determine study characteristics, results, and quality. We graded the strength of the evidence as excellent, good, fair or poor using predetermined criteria. The main outcome measures were evidence of effectiveness and cost of strategies to improve health care quality or reduce disparities in care for racial/ethnic minorities.

**Results:**

Twenty-seven studies met criteria for review. Almost all (n = 26) took place in the primary care setting, and most (n = 19) focused on improving provision of preventive services. Only two studies were designed specifically to meet the needs of racial/ethnic minority patients. All 10 studies that used a provider reminder system for provision of standardized services (mostly preventive) reported favorable outcomes. The following quality improvement strategies demonstrated favorable results but were used in a small number of studies: bypassing the physician to offer preventive services directly to patients (2 of 2 studies favorable), provider education alone (2 of 2 studies favorable), use of a structured questionnaire to assess adolescent health behaviors (1 of 1 study favorable), and use of remote simultaneous translation (1 of 1 study favorable). Interventions employing more than one main strategy were used in 9 studies with inconsistent results. There were limited data on the costs of these strategies, as only one study reported cost data.

**Conclusion:**

There are several promising strategies that may improve health care quality for racial/ethnic minorities, but a lack of studies specifically targeting disease areas and processes of care for which disparities have been previously documented. Further research and funding is needed to evaluate strategies designed to reduce disparities in health care quality for racial/ethnic minorities.

## Background

In recent years, it has become clear that the healthcare system in the United States does not provide the same quality of care for minority populations that it does for the majority white population [1]. The Institute of Medicine (IOM) report "Unequal Treatment" confirmed that racial and ethnic disparities in healthcare are not entirely explained by differences in access, clinical appropriateness, or patient preferences [2]. Additionally, there is increasing evidence that provider behaviors and practice patterns contribute to disparities in care [3] and that healthcare organizational processes also compromise quality [4].

Despite awareness of inequities in healthcare quality, little is known about strategies that have the potential to improve the quality of healthcare for ethnic minority populations. For those interested in quality improvement, there is a need for an evaluation and synthesis of the strategies proven to be effective in bettering the quality of healthcare for minorities. Moreover, it is unknown whether strategies specifically designed to reduce disparities in healthcare between racial/ethnic minorities and whites (in contrast to those simply aimed at improving quality) have been implemented successfully.

The purpose of this study was to systematically review the best evidence to determine the effectiveness and costs of interventions designed to improve the quality of healthcare and/or to reduce disparities for racial/ethnic minorities. Our study focuses on evaluations of interventions aimed at healthcare providers, as recent work suggests provider and organizational factors contribute substantially to the inequities [2]. Furthermore, our study focuses on controlled trials, rather than on evaluations with a weaker study designs, because we were interested to learn which quality improvement strategies had been evaluated most rigorously.

## Methods

### Study design

We conducted a systematic review of the literature, using formal methods of literature identification, selection of relevant articles, data abstraction, quality assessment and synthesis of results, to determine which strategies improve the quality of care for racial/ethnic minorities have been implemented and are effective, and which strategies have been shown to reduce disparities in care [5]. We separately synthesized articles that evaluated the cultural competence education of health professionals, and report those results elsewhere [6].

**Figure 1:**
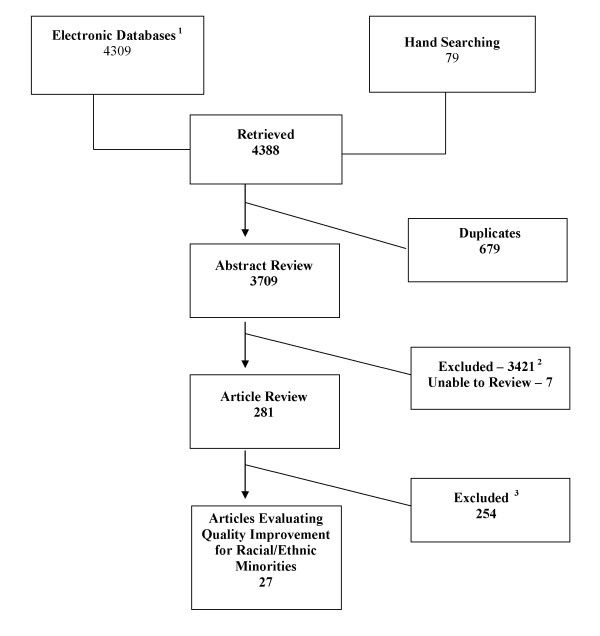
**Summary of literature search and review for eligible articles (# indicates citations or articles at each step)**. Notes: ^1 ^Search strategy used for PubMed was as follows: ((minority groups [mh] OR ethnic groups [mh] OR urban health [mh] OR urban population [mh] OR minority [tiab] OR urban [tiab] OR inner-city [tiab] OR black* [tiab] OR african american* [tiab] OR mexican* [tiab] OR native* [tiab] OR indian* [tiab] OR latina [tiab] OR latino [tiab]) AND (nurs* [tiab] OR physician* [tiab] OR health professional* [tiab] OR health care provider* [tiab] OR health personnel [mh]) AND (randomized controlled trial [pt] OR controlled clinical trial [pt] OR randomized controlled trials [mh] OR random allocation [mh] OR double-blind method [mh] OR single-blind method [mh]) NOT (animal [mh] NOT human [mh])) OR ((cultura* [tiab] OR multicultural [tiab] OR transcultural [tiab] OR divers* [tiab] OR cultural diversity [mh] OR transcultural nursing [mh] OR ethnic [tw] OR minority [tw]) AND (competen* [tiab] OR sensitiv* [tiab] OR attitude* [tiab] OR experience [tiab] OR knowledge [tiab]) AND (education [mh] OR ed [sh] OR educat* [tiab] OR train* [tiab] OR curriculum [tiab]) AND (nurs* [tiab] OR physician* [tiab] OR health professional* [tiab] OR health care provider* [tiab] OR student* [tiab])) AND eng [la] AND 1980:2003 [dp] NOT review [pt] ^2 ^The most common reasons for exclusion were that the abstract was not relevant to minority health (n = 1876) and that the article did not describe an intervention (n = 1655).

In February 2003, we searched: (1) MEDLINE, (2) the Cochrane CENTRAL Register of Controlled Trials (Issue 1, 2003), (3) EMBASE, (4) the specialized register of Effective Practice and Organization of Care Cochrane Review Group (EPOC), (5) the Research and Development Resource Base in Continuing Medical Education (RDRB/CME) and (6) the Cumulative Index of Nursing and Allied Health Literature (CINAHL^®^. We designed search strategies, specific to each database, to maximize sensitivity. Initially, we developed a core strategy for MEDLINE^®^, accessed via PubMed, based on an analysis of the Medical Subject Headings (MeSH) and text words of key articles identified a priori (see footnote in Figure 1). The PubMed strategy formed the basis for the strategies developed for the other electronic databases [5].

In addition to electronic searching, we identified priority journals that provided the largest number of citations in the electronic searching, and we scanned the tables of content from February 1, 2003 through June 15, 2003. We also scanned the reference lists of key review articles that were identified in our electronic searching and all articles eligible for our report.

The results of the searches were imported into ProCite, a reference management software program. This database was used to store citations, track search results and sources, and track the abstract and article review process.

### Eligibility criteria

The following criteria were used to exclude articles: published prior to 1980, not in English, did not include human data, contained no original data, a meeting abstract only (no full article for review), not specific to minority health (overall patient population less than 50% minority or no subgroup analysis), did not occur in the United States, no intervention, intervention not targeted to healthcare providers or organizations, no evaluation of the intervention, not a randomized controlled trial or concurrent controlled trial, or article did not apply to any of the study questions. The term 'minority' was defined as all non-Caucasian or non-white racial and ethnic categories, including African American, Hispanic, American Indian/Alaskan Native, and Asian/Pacific Islander.

Two team members independently reviewed the title and abstract of all citations identified through the literature search. When reviewers agreed that an article was eligible or a decision regarding eligibility could not be made because of insufficient information, the full article was retrieved for review. When reviewers disagreed on eligibility, citations were returned for adjudication.

### Article review

We developed standardized review forms to (1) confirm eligibility for full article review, (2) assess study and participant characteristics and (3) extract the relevant data to address the study questions. The forms were developed through an iterative process that included the review of forms used for previous systematic reviews, discussions among team members and experts, and pilot testing.

For each eligible study, we abstracted data regarding the targeted health care providers and setting, the patient population, the intervention objectives and methods, the measured outcomes, and the intervention effects. We used a conceptual framework adapted from Cooper et al. [7] to categorize the types of outcomes into the following categories: use of services, quality of providers, appropriateness of care, efficacy of treatment, patient adherence, patient ratings of care, and health status. For example, 'quality of providers' included such outcomes as provider knowledge and communication behaviors. We also designed several questions to assess methodological rigor of the studies in each of five domains: representativeness, bias/confounding, intervention description, outcome assessment, and analysis.

We used a serial review process such that a primary reviewer completed the quality assessment and data abstraction forms and a second reviewer, after reading the article, checked each item on the form for completeness and accuracy. Differences between primary and secondary reviewers were resolved by adjudication and, when necessary, consultation with the entire team of reviewers.

### Evidence grading

Once all articles were reviewed and data were synthesized, the strength of the evidence supporting each question was graded into four categories (Grades A-D) based on its quality, quantity, and consistency [8]. To meet the *quality *criteria for Grade A, there must have been at least one randomized controlled trial. To meet the quality criteria for Grade B, C, or D there must have been at least one controlled trial (not necessarily randomized).

For *quantity *of studies, there had to be at least 4 studies available in the literature to meet criteria for Grade A, 3 studies to meet criteria for Grade B, 2 studies to meet criteria for Grade C, or fewer than 2 studies to meet criteria for Grade D. For *consistency*, the results of the studies had to be consistent (either beneficial or harmful results in same direction across studies) to meet criteria for Grade A, reasonably consistent to meet criteria for Grade B (most study results in the same direction), and inconsistent to meet criteria for Grade C. If there were too few studies to judge the consistency (i.e. fewer than three studies), the strength of evidence supporting the question was given a grade of D. The grading of the evidence was discussed at a team meeting and consensus was reached on each criterion. The evidence received a final "grade" that reflected the lowest rank on each of the three criteria (quality, quantity and consistency).

## Results

### Literature search and review process

Results of the literature search and review process are summarized in Figure 1. Of the 4,833 citations identified through search processes, 3,709 were uniquely identified. Of these, 281 were eligible for full article review. After review, 254 were excluded and a total of 27 articles met eligibility criteria [9-35].

### Description of studies

Key study characteristics are summarized in Table 1 and a more detailed description of these characteristics are presented in a supplementary table [See Additional file 1].

### Publication dates and study designs

Of the 27 studies eligible for review, only three studies were published before 1990 [9-11], 20 were published between 1990 and 1999 [12-31], and four were published after 2000 [32-35]. All studies were randomized controlled trials (n = 20) or concurrent controlled trials (n = 7).

### Clinical areas

The majority of articles were in the area of prevention. The most common types of preventive services targeted by the interventions were breast cancer screening (n = 11) and cervical cancer screening (n = 6). There were three studies published in the area of mental health (two in depression and one in alcohol abuse) and one each in the area of chronic kidney disease, asthma, acute respiratory tract infections, emergency medical systems, and advance directives.

### Subjects and settings

Almost all studies were targeted at physicians. Two studies were not targeted at physicians: one was directed solely at nurses and medical assistants and the other was aimed at emergency medical personnel. The specialty of the targeted physicians was most often internal medicine (n = 7), but there were also general primary care (n = 3), pediatrics (n = 3), family medicine (n = 2), adolescent medicine (n = 1), and one or more of the above (n = 9). The interventions targeted practicing professionals (n = 15), professionals in training (n = 6), or both (n = 6). All interventions occurred in the outpatient setting.

Most studies had more than 50 percent African American patients (n = 19) [9,10,12-17,19,21,23,25-28,30-32,35]; only two had patient populations that were specified as more than 50 percent Hispanic [18,20]. and none had more than 50 percent Asian/Pacific Islander patients or more than 50 percent American Indian/Alaska Native patients. The remaining six studies had diverse groups (but no more than 50 percent in any one racial/ethnic category) [11,22,24,29,33,34].

### Intervention methods

All studies had a provider-targeted intervention, and most studies used more than one provider intervention method. The *primary *intervention was a tracking/reminder system in ten studies (in which the clinician is given an automated reminder that a particular service might be due), provider education in two studies, bypassing the physician using nurse/nurse practitioners to offer standardized services directly to patients in two studies, provision of a structured patient questionnaire to facilitate patient-provider communication about patient health behaviors in one study, use of remote simultaneous translation (in which the interpreter translates simultaneously with the speaker but is not in the exam room) in one study, use of subspecialty consultation in one study, and use of defibrillators on emergency medical vehicles in one study. There were nine studies that used more than one main method (multifaceted). Approximately half (n = 14) of the studies had a patient intervention component, although these studies varied in whether the patient intervention was provided in addition to the provider intervention (n = 10) or compared to the provider intervention (n = 4). The intervention was tailored specifically for racial/ethnic minorities in only two studies [18,34].

### Outcome assessment

The most common outcomes were related to healthcare process: use of services (7 studies, 13 outcomes), appropriateness of care (18 studies, 43 outcomes), quality of providers (9 studies, 30 outcomes), patient adherence (4 studies, 9 outcomes), and efficacy of treatment (1 study, 1 outcome). Patient health status (7 studies, 21 outcomes), and patient ratings of care (3 studies, 3 outcomes) were also measured.

### Quality of reviewed studies

Selected aspects of quality are summarized in Table 2. In general, the studies provided a thorough description of the subjects, setting and intervention strategy. Although there were 20 randomized controlled trials, the randomization was considered adequate (in that investigators could not predict assignment) in only 11 studies. Although all studies used objective methods to evaluate outcomes, only nine of 27 studies had blinded outcome assessment, and only 13 of 27 studies performed a pre- and a post-intervention evaluation. Approximately half reported the numbers for and reasons for non-inclusion in the study analysis, and almost all performed a complete statistical analysis (including the magnitude of difference between groups, an index of variability, and a test statistic).

### Effectiveness of interventions to improve the quality of healthcare for minorities

Results are summarized in Table 3. Appendix A provides further detail for each study [See Additional file 2]. Each study used a unique combination of intervention methods in a variety of settings and patient populations. However, for the purpose of synthesis, we have identified the *main *intervention method. It should be noted that the categorization of the main intervention method is a simplification of what was often a complex intervention strategy.

### Tracking/reminder systems

Ten studies used tracking and/or reminder systems to improve quality of care. Of these, two were in adult general prevention [9,10], six were in adult cancer screening [10,15,19,21,25,35], one in tobacco cessation [30], and one was in end-of-life care (completion of advance directives) [31]. All ten studies demonstrated positive outcomes, primarily in the appropriateness of care (such as provision of preventive care, tobacco cessation counseling, or advance directive counseling) category. Overall, there is excellent evidence supporting the use of tracking/reminder systems aimed at providers of racial/ethnic minority patients (Evidence Grade A).

### Multifaceted interventions

Nine studies used an intervention that we characterize as multifaceted, meaning that there two or more (usually more) main intervention methods [14,20,22,24,27-29,33,34]. Examples of these types of intervention are detailed in the supplementary table [See Additional file 1]. Two of these interventions were in adult cancer screening [28,29], one in tobacco cessation [27], one in cholesterol reduction [24], two in depression [14,34], one in alcohol cessation [20], one in acute upper respiratory tract infections [33], and one in asthma [22]. Outcomes of these studies are mixed, with most studies showing improvements in one or two (but not all) outcomes measured. Overall, there is fair evidence supporting the use of multifaceted interventions aimed at providers of racial/ethnic minority patients (Evidence Grade C).

### Bypass the physician

Two studies (both in adult cancer screening) bypassed the physician and had either a nurse or a nurse practitioner offer screening directly to patients, and both studies demonstrated improvements in the provision of preventive services to patients [12,23]. Overall, there is fair evidence (favorable results but only two studies) supporting the use of bypassing the providers of racial/ethnic minority patients to offer standardized services directly to patients (Evidence Grade C).

### Provider education

Two studies used provider education as the main intervention strategy, one in the area of adult general prevention [17] and one in prevention of injuries in children [32]. Both studies found improvements in provider counseling behaviors [17,32], but one measured and did not find any positive effect of the intervention on parental knowledge of injury prevention or parental adherence to provider advice [32]. Overall, there is fair evidence supporting the use of provider education aimed at providers of racial/ethnic minority patients (Evidence Grade C).

### Use of Safe Times Questionnaire (STQ)

One study (in the area of prevention for children) used a structured questionnaire to assess adolescent health behaviors, provided the results of the questionnaire to the provider, and demonstrated a positive impact on provider counseling behaviors [16]. However, because of the insufficient number of studies using this method, there is poor evidence supporting the use of structured questionnaires for racial/ethnic minority patients (Evidence Grade D).

### Use of Remote Simultaneous Translation (RST)

One study compared the accuracy of translation and quality of patient-physician communication by using remote simultaneous and proximate consecutive interpretation and found fewer translation errors and enhanced patient and physician satisfaction by using the RST method [18]. However, because of the insufficient number of studies using this method, there is poor evidence supporting the use of remote simultaneous translation for racial/ethnic minority patients (Evidence Grade D).

### Use of specialty consultation

One study evaluated the use of nephrology consults for patients with chronic kidney disease and found no effect on healthcare process or patient outcomes [26]. However, because of the insufficient number of studies using this method, there is poor evidence supporting the use of specialty consults to improve the quality of care for racial/ethnic minority patients (Evidence Grade D).

### Use of defibrillators on emergency medical services

One study evaluated the use of defibrillators on emergency medical services and found no effect on patient outcomes [13]. Overall, there is poor evidence supporting the use of defibrillators on emergency medical services (Evidence Grade D).

### Effectiveness of interventions to reduce disparities

Only one study specifically addressed the question of whether an intervention could reduce disparities in healthcare quality between ethnic minority and white persons [34]. The study, which evaluated the impact of two different culturally tailored interventions to improve the quality of depression care compared with a control group that received no intervention, had mixed results. There was no differential effect of the interventions on healthcare process for white versus ethnic minority patients; all patients (African-American, Latino, and white) in the interventions groups were more likely than patients in the control group to receive appropriate therapy. However, there was a mixed effect on health outcomes: there were improvements for African-American and Latino patients in the rate of depression remission compared to controls (with no improvement for white patients), but there were no improvements for African-American and Latino patients in the intervention groups in employment rates compared with controls (with improvement for white patients). Overall, there is poor evidence to determine which interventions might reduce disparities between racial/ethnic minority patients and majority patients (Evidence Grade D).

### Costs of quality improvement for racial/ethnic minorities

Only one study reported on the costs of an intervention aimed at improving the quality of healthcare for racial/ethnic minority persons [26]. This study, which provided case management and nephrology consultation for patients with chronic renal insufficiency, estimated that it cost a minimum of US $89,355 yearly in 1998 (or $484 per intervention patient), but it found no health benefits to participants. Overall, there is poor evidence to determine the cost of strategies to improve the quality of care for racial/ethnic minorities (Evidence Grade D).

## Discussion

We rigorously evaluated strategies to improve the quality of care for racial/ethnic minority patients in a select group of studies. Almost all the interventions occurred in the primary care setting, and most focused on the provision of preventive services. Only two of the studies were specifically targeted at the needs of racial/ethnic minority patients [18,34]; the remaining studies were generic quality improvement strategies. Despite this, there is excellent evidence that provider tracking/reminder systems are effective in improving quality of care for racial/ethnic minority patients (Evidence Grade A), fair evidence that multifaceted interventions, provider education interventions, and interventions that bypass the physician to offer screening services to racial/ethnic minority patients can improve quality of care (Evidence Grade C), and poor evidence for the use of any other of the studied intervention strategies (Evidence Grade D).

Tracking and reminder systems were effective in improving rates of standardized services such as cancer screening [10,15,19,21,25,35] and advance directive completion [31]. Although these do not represent all clinical areas for which disparities in care have been documented and thus may hold little potential for addressing overall disparities, the strategy appears effective in improving the process of care for racial/ethnic minorities. In some studies, though, some of the processes of care targeted by tracking and reminder systems were not evidence-based practices for any patient population (for example, oral cavity exams or breast self-examinations for cancer screening) and would therefore be unlikely to improve the quality of care or reduce disparities for racial/ethnic minority patients.

Our review identifies important gaps in knowledge that provide a focus for future research. Regarding strategies worthy of further study, there were several types of interventions with favorable results, but employed in only one or two studies each, thus receiving a grade of either D or C. These are: (1) bypassing the physician to offer standardized services directly to patients [12,23], (2) use of remote simultaneous translation for patients with limited English proficiency [18] and (3) the use of a structured questionnaire (Safe Times Questionnaire) for health behaviors risk assessment in adolescents [16]. Of these, the study that evaluated remote simultaneous translation is particularly germane to the needs of some racial/ethnic minorities, and could have widespread impact if the results are replicated in other studies. Also, both of the studies evaluating provider education yielded favorable results [17,32]; however, other studies have suggested that passive educational interventions are not optimal [37].

We found poor evidence to determine which strategies are most effective in reducing disparities between minority and white populations (Evidence Grade D). The only study that was specifically designed to do this had mixed results; with improvements in only one of the two outcomes assessed [34]. This represents a critical gap in the literature. More research is needed that is designed specifically to reduce disparities in healthcare quality, for example, research that targets specific diseases (e.g. cardiovascular disease, diabetes, HIV) and healthcare processes known to be a source of racial/ethnic disparities. It may be necessary to distinguish between interventions aimed at improving the quality of care for all persons and those aimed at improving quality of care for racial/ethnic minority populations specifically (such as reducing provider bias or improving intercultural communication skills). When generic quality improvement interventions are done in diverse populations, subgroup analyses that evaluate the effect of the interventions in racial/ethnic minority patients would increase our understanding the effect on equity of treatment.

Many organizations have limited resources to accomplish the goal of improving minority health care quality. Our review found poor evidence to determine the costs of strategies to improve care and reduce disparities for minority populations (Evidence Grade D). Only one study included an estimate of cost. In order to make resource allocation decisions for ethnic minority patient populations in the future, data on the costs of these interventions will be critical.

Additional knowledge gaps to be filled by future research were related to targeted groups, settings, and health outcome assessments. First, there are very few quality improvement interventions that were completed in Hispanic populations and none in American Indians/Alaska Natives or in Asians/Pacific Islanders. Second, almost all studies were done in the primary care setting. Insofar as disparities have been documented in other settings, more studies may be needed in acute care and specialty settings. Finally, few studies measured patient outcomes; most measured healthcare process. This limitation would not be as important if all studies had targeted processes of care that were evidence-based and more closely linked to patient outcomes. Studies need to include patient outcomes, have longer follow-up to determine the sustainability of intervention effects, and link process of care to health outcomes.

There were some challenges in synthesizing this body of literature. First, studies that used multifaceted interventions and did not examine separate components, making it difficult to know exactly what resulted in the beneficial effect observed. Second, each study used slightly different intervention methods, making generalizations across studies difficult. Because no two studies used exactly the same intervention and evaluation strategy, there is a need to replicate and evaluate promising intervention strategies in different healthcare settings and organizations. In addition, multifaceted interventions should be evaluated in such a way so as to distinguish which specific piece of the intervention was most effective.

This review has several limitations. First, eligibility was limited to English language and to published reports of studies. Although our resources did not permit extensive searching of the non-English language and unpublished literature, recent work has suggested that results of reviews with these limits do not differ substantially from reviews with no such limits [38]. There is, however, a possibility of publication bias, in that studies with demonstrated benefit are more likely to be published than those with no benefit. This is a limitation of any systematic review.

Eligibility was also limited to articles published after 1980, and to studies conducted in the United States. There may have been other promising interventions conducted in other countries that are not reflected in this report. Only randomized controlled trials and concurrent controlled trials were included. Although researchers have recommended the use of experimental designs with control groups to evaluate interventions to reduce disparities whenever possible [7], there have been other worthwhile interventions that have been evaluated with other study designs [39]. Our review, which found a paucity of rigorous clinical trials, suggests that other types of studies should be considered by those interested in designing interventions to reduce disparities. To the extent that other QI strategies are used without a sound evidence base, it may be unfair to hold interventions designed to reduce disparities to a different standard. On the other hand, it is a worthwhile goal that all QI interventions have demonstrated effectiveness. Our review only included interventions targeting providers; interventions directly targeting patients may also be promising strategies to improve the quality of care and reduce racial/ethnic disparities, but they are not reflected in this report.

## Conclusion

In conclusion, there are several promising strategies that may improve health care quality for racial/ethnic minorities, but a lack of studies specifically targeting the disease areas and the processes of care for which disparities have been previously documented. This body of research should be a national priority as it will aid in clinical decision-making and health care resource allocation.

## Competing interests

The author(s) declare that they have no competing interests.

## Authors' contributions

MCB, TLG, EGP, KR, AG, AP, CS, MJ, EBB, NRP, and LAC were involved in the conceptualization of the research question. MCB, KR, EBB, NRP, and LAC took responsibility for overall coordination of project. KR developed the literature search strategy. All authors reviewed abstracts and articles. MCB, KR, MJ, and CF organized and synthesized the data. MCB drafted the manuscript. All authors read and approved the manuscript

## Pre-publication history

The pre-publication history for this paper can be accessed here:



## Supplementary Material

Additional File 1 Selected characteristics of the 27 studies aimed at improving healthcare quality for racial/ethnic minorities Click here for file

Additional File 2 Results of 27 studies aimed at improving the quality of healthcare for racial/ethnic minorities Click here for file

## Figures and Tables

**Table 1:** Summary of 27 studies evaluating interventions to improve the quality of healthcare for racial/ethnic minorities

		# studies
**Publication Date**	1980–1989	3
	1990–1999	20
	2000–2003	4
		
**Study Design**	RCT	20
	CCT	7
		
**Study Setting**	Hospital Outpatient Setting	14
	Community Health Center	4
	Group Practice	2
	Community	1
	>one of the above	6
		
**Clinical Areas**	Prevention	19
	Mental Health	3
	Chronic kidney Disease	1
	Asthma	1
	Acute Respiratory Tract Infections	1
	Emergency Medical Systems	1
	End of Life Care	1
		
**Targeted Health Providers**	Physicians	17
	Non-physicians	2
	Physicians and Non-physicians	8
		
**Patient Ethnicity**	>50% African American	19
	>50% Latino	2
	>50% Asian/Pacific Islander	0
	>50% American Indian/Alaskan Native	0
		
**Main Provider Intervention**	Tracking/Reminder	10
	Provider Education	2
	Bypass Physician	2
	Structured Patient Questionnaire	1
	Remote Simultaneous Translation	1
	Subspecialty Consult	1
	Defibrillators on Emergency Vehicles	1
	> one of the above (Multifaceted)	9
		
**Patient Intervention**	Present	14
	Absent	13

**Table 2:** Summary of selected aspects of study quality for the 27 studies aimed at improving quality of health care for racial/ethnic minorities

**Selected Quality Assessment Domains and Items**	**Articles (Total N = 27)**	
	**N**	**%**

**Representativeness**		
- Healthcare providers clearly described?	20	74%
**Bias/confounding**		
- Adequate comparison group?	26	96%
- Adequate randomization?	11	41%
**Intervention description**		
- Complete description (able to replicate)?	24	89%
**Outcome assessment**		
- Blinding?	9	67%
- Pre- and post-test?	13	48%
**Analysis**		
- Numbers and reasons of non-inclusion?	18	67%
- Complete statistical analysis?	24	89%

**Table 3:** Summary of interventions, clinical areas, and outcomes of 27 studies aimed at improving quality of health care for racial/ethnic minorities

**Intervention Type**^1^	**Clinical Area (references)**	**Total # Studies**	**Overall Outcome Assessment across Studies**	**Rating of Evidence**
Tracking/Reminders	General Adult Prevention (9,10)	10	All studies reported favorable outcomes.	A
	Cancer Screening (10,15,19,21,25,35)			
	Tobacco Cessation (30)			
	End of Life (31)			
Multifaceted	Adult Cancer Screening (28,29)	9	Although almost all studies reported some favorable impact on one or more outcome type, results were inconsistent across studies	C
	Cholesterol Reduction(24)			
	Tobacco Cessation (27)			
	Depression (14,34)			
	Alcohol Cessation (20)			
	Asthma (22)			
	Upper Respiratory Tract Infections (33)			
Bypass MD	Cancer Screening (12,23)	2	Both studies reported favorable outcomes in the provision of preventive services to patients	C
Provider Education	Adult General Prevention (17)	2	Both studies reported favorable impact on provider counseling behaviors.	C
	Child Injury Prevention (32)			
Use of STQ^2^	Adolescent Health Risks (16)	1	One study reported favorable impact on provider counseling behavior.	D
Use of RST^3^	Well Baby Care (18)	1	One study reported favorable outcomes on accuracy of translation and provider/patient satisfaction.	D
Use of Specialty Consult	Chronic Renal Disease (26)	1	One study did not demonstrate improvement.	D
Use of Defibrillators	Emergency Medicine (13)	1	One study did not demonstrate improvement.	D

^1 ^Note that characterization of interventions for purposes of synthesis reflects a simplification of employed methods. Consult Supplementary Table for details on methods for individual studies [See Additional file 1].

^2 ^STQ = safe times questionnaire

^3 ^RST = remote simultaneous translation
