# Supplementary material for: Improving health care quality for racial/ethnic minorities: a systematic review of the best evidence regarding provider and organization interventions
Source: BMC Public Health. 2006 Apr 24;6:104. doi: 10.1186/1471-2458-6-104 (PMC1525173; doi:10.1186/1471-2458-6-104)
Supplement: Additional File 1 — Selected characteristics of the 27 studies aimed at improving healthcare quality for racial/ethnic minorities [file 1471-2458-6-104-S1.doc]

| **SUPPLEMENTARY TABLE** Selected characteristics of the 27 studies aimed at improving healthcare quality for racial/ethnic minorities | | | | | | | |
| --- | --- | --- | --- | --- | --- | --- | --- |
| **Study** | **Targeted Healthcare Providers/ Levels of Training/ Number of Providers** | **Study Design ^a^** | **Study Setting** | **Group** | **Patient Race/ Ethnicity** | **Provider  Interventions** | **Patient  Interventions** |
|  |  |  |  |  |  |  |  |
| Gemson 1995 | Physician Resident/fellow, Professional n=254 | CCT | Hospital Outpatient | Control  n=471 | 93% AA, 5% L | No Interventions |  |
|  |  |  |  | Intervention n=529 | 92% AA, 5% L | Educ., pads of prevention prescription forms |  |
| McDonald  1984 | Physician, nurse clinicians Resident/fellow, Professional n=126 | RCT | Hospital Outpatient | Control   n=NS |  | No Interventions |  |
|  |  |  |  | Intervention n=NS |  | TRS |  |
| Turner 1989 | Physician, Resident/fellow n=34 | CCT | Hospital Outpatient | Pt. intervention n=86 |  |  | Questionnaire of status of preventive services before primary care visit |
|  |  |  |  | Phys. and Pt. interventions n=64 |  | TRS | Questionnaire of status of preventive services before primary care visit |
|  |  |  |  | Phys. intervention  n=103 |  | TRS |  |
| Burack 1994 | Nurse, Physician Professional n=25 | RCT | Community Health Clinic, Hospital Outpatient, HMO | Full intervention n=1382 |  | Educ., TRS | Elimination of out-of-Pocket expenses for Pts., post-card and telephone follow-up |
|  |  |  |  | Limited intervention n=1343 |  | Educ. | Elimination of out-of-pocket expenses for patients |
| Burack 1996 | Physician Professional n=20 | RCT | Group Practice, HMO | Control  n=596 |  | No Interventions |  |
|  |  |  |  | Pt. reminders n=592 |  |  | Pt. reminder letters |
|  |  |  |  | Phys. and Pt. reminders  n=590 |  | TRS | Pt. reminder letters |
|  |  |  |  | Phys. reminders n=590 |  | TRS |  |
| Burack 1997 | Physician Professional  n=not applicable | RCT | Community Health Clinic, HMO | Full intervention n=1413 |  | Educ., TRS |  |
|  |  |  |  | Limited intervention n=1413 |  | Educ. |  |
| Burack 1998 | Physician Professional n=20 | RCT | Group Practice | Control   n=964 |  | No Interventions |  |
|  |  |  |  | Pt. reminders n=964 |  |  | Pt. reminder letters |
|  |  |  |  | Phys. and Pt. reminder  n=960 |  | TRS | Pt. reminder letters |
|  |  |  |  | Phys. reminders n=960 |  | TRS |  |
| Burack 2003 | Physician Professional n=20 | RCT | Home/ Community, HMO | Mammogram only reminder group n=1228 |  | TRS | Pt. reminder letter |
|  |  |  |  | Pap smear and mammogram reminder group n=1243 |  | TRS | Pt. reminder letter |
| Chambers 1989 | Physician Resident/fellow, Professional n=30 | RCT | Hospital Outpatient | Control n=623 | 31% W | No Interventions |  |
|  |  |  |  | Intervention n=639 | 28% W | TRS |  |
| Dietrich  1998 | Nurse, Physician, Office staff, Clinical directors Professional n=NS | RCT | Community Health Clinic | Control n=1267 | 23% AA, 22% W, 26% L | No Interventions |  |
|  |  |  |  | Intervention n=1381 | 31% AA, 22% W, 22% L | Multifaceted: Educ., TRS, preventive care flow sheets, advice to center leaders, external chart identifiers | Patient-held health diaries, Pt. educ. materials |
| Mandelblatt 1993 | Nurse, Physician Resident/fellow, Professional n=NS | CCT | Hospital Outpatient | Control  n=NS | 82% AA, 15% W, 2% L | TRS |  |
|  |  |  |  | Intervention n=NS | 93% AA, 1% W, 1% L | Screening offered directly to Pt. by nurse practitioner | Pt. counseling and screening offered by nurse practitioner |
| Manfredi 1998 | Physician Professional n=NS | RCT | Group & Solo Practices, HMO, Community of free standing clinics | Control n=42230 |  | Flow sheets supplied but not actively encouraged |  |
|  |  |  |  | Intervention n=52392 |  | Multifaceted: Educ., TRS, Audit & Feedback, guidelines and quality assurance procedures | Health maintenance cards with from HMO |
| McCarthy 1997 | Nurse, Medical assistant Professional n=NS | CCT | Hospital Outpatient | Control (Clinic A) n=2560 | 79% AA, 18% W | No Interventions |  |
|  |  |  |  | Control (Clinic B) n=2124 | 73% AA, 24% W | No Interventions |  |
|  |  |  |  | Intervention n=1250 | 82% AA, 15% W | Educ., Nurses and medical assistants screened all patients to ensure mammography and initiated referrals for patients |  |
| Ahluwalia 1999 | Physician Resident/fellow n=45 | CCT | Hospital Outpatient | Control  n=NS | 100% AA | No Interventions |  |
|  |  |  |  | Intervention n=NS | 100% AA | TRS, smoking stamp which Phys. needed to mark as never, former, or current |  |
|  |  |  |  |  |  |  |  |
| Allen 1998 | Physician Resident/fellow n=158 | RCT | Hospital Outpatient | Control  n=571 | 100% AA | No Interventions |  |
|  |  |  |  | Intervention n=515 | 100% AA | Multifaceted: Educ., Reward Incentive, summary sheet of Pt's smoking history on outside of medical record | Written material given to Pts. about smoking cessation |
| Keyserling 1997 | Physician, Nurse practit, Physician assistants Professional n=42 | RCT | Community Health Clinic | Control  n=188 | 40% AA, 47% W, 12% AI/NA | No Interventions |  |
|  |  |  |  | Intervention n=184 | 39% AA, 50% W, 11% AI/NA | Multifaceted: Educ., TRS, nutritionist completed summary letter to clinician urging drug treatment for those with no response to diet therapy after 7 months | Pt. educ. materials |
| Schubiner 1994 | Physician Pre-professional training, Resident/ fellow n=NS | RCT | Community Health Clinic | Control (Interview)  n=NS |  | Educ. |  |
|  |  |  |  | Intervention (STQ) n=NS |  | Educ., provision of Safe Times Questionnaire completed by Pt. |  |
|  |  |  |  |  |  |  |  |
| Gielen 2001 | Physician Resident/fellow n=31 | RCT | Hospital Outpatient | Control  n=76 |  | Educ. |  |
|  |  |  |  | Intervention n=120 |  | Educ. |  |
| Hornberger 1996 | Physician Professional n=4 | RCT | Hospital Outpatient | Proximate consecutive translation n=NS | 100% L | Proximate consecutive translation of Pt. and Phys. statements by an interpreter in the examination room |  |
|  |  |  |  | Remote simultaneous translation n=NS | 100% L | Remote simultaneous translation of Pt. and Phys. statements by interpreter in another location |  |
| Burge 1997 | Physician Resident/fellow n=NS | RCT | Hospital Outpatient | Control  n=NS | 100% L | No Interventions |  |
|  |  |  |  | Pt. intervention n=NS | 100% L |  | Psychoeducation (9 hours) |
|  |  |  |  | Phys. and Pt. intervention n=NS | 100% L | Multifaceted: Educ., TRS, protocols/guidelines | Psychoeducation (9 hours) |
|  |  |  |  | Phys. intervention n=NS | 100% L | Multifaceted: Educ., TRS, protocols/guidelines |  |
| Callahan 1994 | Physician Professional n=103 | RCT | Group Practice | Control  n=100 | 53% AA | Educ. |  |
|  |  |  |  | Intervention  n=75 | 50% AA | Multifaceted: Educ., protocols/guidelines, 3 additional primary care visits with depressed Pts. |  |
| Miranda 2003 | Nurse, Physician, Nurse pract Professional n=181 | RCT | Group Practice, HMO Primary Care Practice | Behavioral therapists  n=NS |  | Multifaceted: Educ., resources to support costs | Culturally tailored materials and Cognitive Behavioral Therapy for 8-12 sessions |
|  |  |  |  | Control  n=NS |  |  |  |
|  |  |  |  | Nurse follow-up n=NS |  | Multifaceted: Educ., resources to support costs | Culturally tailored materials and nurse follow-up for 6 to 12 months |
| Harris 2003 | Physician, Nurse pract Professional n=42 | CCT | Hospital Outpatient | Baseline control group  n=671 | 16% AA, 39% W, 42% L | No Interventions |  |
|  |  |  |  | Full intervention n=393 | 9% AA, 35% W, 41% L | Multifaceted: Educ., protocols/ guidelines, posters in exam rooms | Posters, computerized Educ. |
|  |  |  |  | Limited intervention n=805 | 12% AA, 37% W, 48% L | Multifaceted: Educ., protocols/ guidelines, posters in exam rooms | Posters |
| Evans 1997 | Nurse, Physician, Clerical staff, Laboratory technicians, Public health assistants Professional n=134 | RCT | Community Health Clinic | Control  n=NS | 46% AA, 34% L | Protocols/ guidelines, access to new medicines |  |
|  |  |  |  | Intervention n=NS | 44% AA, 33% L | Multifaceted: Educ., protocols/guidelines, access to new medicines, monthly visits by nurse-educator to clinic, consultation (telephone) available with "expert" |  |
| Harris 1998 | Physician Resident/fellow, Professional n=not applicable | RCT | Hospital Outpatient | Control n=231 | 80% AA | No Interventions |  |
|  |  |  |  | Intervention n=206 | 81% AA | Letter to primary care Phys. including summary of actions taken by consultant nephrologist, suggestions for future care, and summary of clinic visit | Consultation with nephrologist |
| Kellermann 1993 | Firefighters Professional n=40 | CCT | Home/Community | CPR control n=432 | 59% AA | No Interventions |  |
|  |  |  |  | External defibrillators n=447 | 60% AA | Educ. |  |
| Dexter 1998 | Physician Resident/fellow, Professional n=147 | RCT | Hospital Outpatient | Control  n=253 | 60% AA, 39% W | Educ. |  |
|  |  |  |  | Instruction and Proxy Directive n=277 | 56% AA, 44% W | Educ., TRS |  |
|  |  |  |  | Instruction Directive  n=219 | 56% AA, 44% W | Educ., TRS |  |
|  |  |  |  | Proxy Directive n=260 | 49% AA, 51% W | Educ., TRS |  |
| ^a^ CCT= controlled clinical trial; RCT= randomized controlled trial | | | | |  |  |  |
| AA = African American; L = Latino/Hispanic; W = White; AI/NA = American Indian or Native Alaskan | | | | | | |  |
| TRS = Tracking/Reminder System | | |  |  |  |  |  |
|  | | | | | | | |
|  | | | | |  |  |  |
|  | | | | |  |  |  |
|  | | | | |  |  |  |
|  |  |  |  |  |  |  |  |
|  | | | | | |  |  |
|  | | |  |  |  |  |  |
|  | | |  |  |  |  |  |
|  | |  |  |  |  |  |  |
|  | |  |  |  |  |  |  |
