# Supplementary material for: Improving health care quality for racial/ethnic minorities: a systematic review of the best evidence regarding provider and organization interventions
Source: BMC Public Health. 2006 Apr 24;6:104. doi: 10.1186/1471-2458-6-104 (PMC1525173; doi:10.1186/1471-2458-6-104)
Supplement: Additional File 2 — Results of 27 studies aimed at improving the quality of healthcare for racial/ethnic minorities [file 1471-2458-6-104-S2.pdf]

Appendix A: Results of 27 studies aimed at improving the quality of healthcare for racial/ethnic minorities[a]

Study	Outcome	How Measured	Intervention Effect	Summary	
				Between-Group Summary	Within-Group Summary
**PREVENTION, ADULT**					
**Adult, General Prevention**					
Gemson, 1995	Breast self examination counseling	Patient report	Changes in breast self-exam counseling rates received by patients pre and post intervention time period: intervention + 0.12 (p<0.01); control -0.06 (p=0.25).	not available	significant improvement
	Nutrition and weight control counseling	Patient report	Changes in nutrition and weight control counseling rates received by patients pre and post intervention time period: intervention +0.11 (p=0.02); control -0.05 (p=0.27).	not available	significant improvement
	Prevention knowledge	Provider written test	The overall change in physician knowledge of prevention was significantly greater for the intervention [+2.67 (SD 3.96)] compared to control [+1.58 (SD 3.43)] (p=0.03).	significant improvement	not available
	Exercise counseling	Provider self report	Positive change in physician exercise counseling practices comparing intervention to control (p<0.01).	significant improvement	not available
	Nutrition and weight control counseling	Provider self report	Positive change in physician nutrition and weight control counseling practices comparing intervention to control (p<0.01).	significant improvement	not available
	Smoking cessation counseling	Patient report	Change in receipt of smoking cessation counseling rates pre and post intervention time period: intervention +0.03 (p=0.08), control +0.01 (p=0.4).	not available	improvement
	Breast cancer screening	Provider self report	Positive change in physician breast cancer screening practices comparing intervention to control (p=0.01).	significant improvement	not available
	Smoking cessation counseling	Provider self report	Positive change in physician smoking cessation counseling practices comparing intervention to control (p<0.01).	significant improvement	not available
	Exercise counseling	Patient report	Changes in exercise counseling rates received by patients pre and post intervention time period: intervention + 0.07 (p<0.01); control + 0.03 (p=0.14).	not available	significant improvement

Study	Outcome	How Measured	Intervention Effect	Summary	
				Between-Group Summary	Within-Group Summary
McDonald, 1984	Blood pressure, weight, serum glucose, serum hemoglobin, serum potassium, and blood urea nitrogen	Medical record review	No significant differences between patients assigned to intervention group physicians and control physicians.	no improvement	not available
	Hospitalizations and emergency room visits for patients eligible for pneumococal or influenza vaccines	Review of admission logs	Patients cared for by intervention group physicians had fewer hospitalizations and emergency room visits in the years influenza occurred (p<0.02).	significant improvement	not available
	Overall number of hospitalizations, emergency room visits, or clinic visits	Review of admission logs	No significant overall differences between patients assigned to intervention group physicians and control physicians.	no improvement	not available
	Physician response to computerized reminders	Computer record review	49% of physicians responded to computerized reminders in intervention group compared to 29% in control group (p<0.001).	significant improvement	not available
	Physician response to computerized reminders regarding preventive care	Computer record review	Physicians in intervention group were 2 to 4 times more likely to apply preventive care to their eligible patients than were control group physicians (p<0.005).	significant improvement	not available
Turner, 1989	Guaiac test rates	Medical record review	Guaiac test rates increased from 34.1% to 50% in the physician-reminder group (p<0.05), increased from 32.6% to 42.5% (p=ns) in the patient-reminder group, and increased from 29.7% to 46.1% (p<0.05) in the combined intervention group.	not available	significant improvement
	Rectal exam rates	Medical record review	Rectal exam rates increased from 10.4% to 52.6% in the physician-reminder group (p<0.05), increased from 32.3% to 46.9% (p<0.05) in the patient-reminder group, and increased from 34.1% to 57.1% (p<0.05) in the combined intervention group.	not available	significant improvement
	Pap smear rates	Medical record review	Pap smear rates increased from 20.3% to 33.1% in the physician-reminder group (p=ns), decreased from 29.4% to 27.5% (p=ns) in the patient-reminder group, and increased from 20.5% to 40% (p=ns) in the combined intervention group.	not available	no improvement
	Breast exam rates	Medical record review	Breast exam rates increased from 37.5% to 53.5% in the physician-reminder group (p<0.05), increased from 41.2% to 53.6% (p<0.05) in the patient-reminder group, and increased from 38.3% to 47.8% (p=ns) in the combined intervention group.	not available	significant improvement

Study	Outcome	How Measured	Intervention Effect	Summary	
				Between-Group Summary	Within-Group Summary
	Mammogram rates	Medical record review	Mammogram rates increased from 14.3% to 15% in the physician-reminder group (p=ns), increased from 15% to 46.6% (p<0.05) in the patient-reminder group, and increased from 0% to 4.8% (p=ns) in the combined intervention group.	not available	significant improvement
	Tetanus immunization rates	Medical record review	No change reported in any group.	not available	no improvement
**Adult, Cancer Screening**					
Burack, 1994	Mammogram appointment	Mammogram appointment rates	At each site, full intervention status was associated with significant increase in mammogram appointment rates with absolute increase from 13% to 29% compared to limited intervention.	significant improvement	not available
	Overall use of mammography	Mammogram appointment completion rates	Compared to limited intervention, full intervention was associated with a significant increase in the mammography rate at each of the sites.	significant improvement	not available
	Completion of first mammogram appointment	Completed mammogram appointments	No differences in completion of first mammography appointment between full intervention groups.	not available	not available
Burack, 1996	Primary care visit rates in 1 year	Administrative data	No significant relation between interventions and primary care visitation.	no improvement	not available
	Mammography screening rates (completion in study year)	Administrative data	Mammography rates were higher for the two groups of women assigned to the physician reminder intervention, but only at one of two HMO sites. No significant difference for patient intervention.	improvement	improvement
Burack, 1997	Annual mammography rates	Medical record review; computerized administrative records	At the end of year 2, mammography rates were 44% for full intervention versus 28% for limited intervention at the health department (OR: 1.84, 95% CI:1.40, 2.40) and 45% for full versus 46% for limited at the health maintenance organization (OR: 1.06, 95% CI: 0.08, 1.42).	significant improvement	not available
Burack, 1998	Completion of pap smear	Reports of pap-smear results	Women who received the patient and physician reminders together had 1.23 times the odds of completing pap smear screening (95% CI, 1.01, 1.50) than women who received neither intervention. The pap smear rates for women in the patient only or physician only intervention were not significantly different than the pap smear rates for women who received neither intervention.	significant improvement	not available
	Primary care visits	Administrative data	No significant association of intervention.	no improvement	not available

Study	Outcome	How Measured	Intervention Effect	Summary	
				Between-Group Summary	Within-Group Summary
Burack, 2003	Completing pap smear	Medical record review	Combined reminder treatment associated with increased likelihood of pap smear (30% versus 23%, p=0.007) OR 1.39.	significant improvement	not available
	Completing mammogram	Medical record review	No difference between combined reminder treatment and mammogram only reminder (39% versus 40%).	no improvement	not available
	Visits to gynecology	Medical record review	Combined reminder treatment associated with increased rate of visits (34% versus 29%, p=0.001).	significant improvement	not available
	Visits to primary care physician	Medical record review	No difference between groups.	no improvement	not available
Chambers, 1989	Mammography rates	Medical record review	At the end of the study period, 27% of the experimental group were up-to-date as compared with 21% of the control group (p=0.011).	significant improvement	not available
Dietrich, 1998	Mammography rates in females over 50	Medical record review	Both interventions and control groups showed increased rates, 58% to 65% in the intervention group (p=0.008) versus 59% to 64% in the control group (p=0.02).	no improvement	improvement
	Oral cavity exam	Medical record review	Both intervention and control groups showed significantly increased rates, 67% to 78% in the intervention group versus 73% to 83% in the control group (p<0.001).	no improvement	significant improvement
	Rates of clinical breast exams in all females	Medical record review	Both intervention and control groups showed increased rates, 50% to 63% in the intervention group versus 55% to 59% in the control group (significant in both groups).	no improvement	significant improvement
	Rates of pap test in all females	Medical record review	Intervention showed no change in rates, changing from 52% to 55%, while the control group increased rates from 43% to 62% (p<0.01)	no improvement	improvement
	Fecal occult blood test	Medical record review	Both intervention and control groups increased rates, 9% to 19% in the intervention group versus 7% to 19% in the control group (not significant in either group).	no improvement	improvement
	Digital rectal exam	Medical record review	Both intervention and control groups showed increased rates, 35% to 41% in the intervention group (not significant) versus 34% to 49% in the control group (p=0.03).	no improvement	improvement
	Rates of breast self-exam advice in all females	Medical record review	Both interventions and control groups showed increased rates, 4% to 13% in the intervention group (p<0.001) versus 3% to 8% in the control group (not significant). Intervention showed a greater increase than control (p=0.009).	significant improvement	significant improvement
	Sigmoidoscopy	Medical record review	Neither intervention or control arm increased rates, 3% to 3% in the intervention group versus 2% to 2% in the control group.	no improvement	no improvement
Mandelblatt, 1993	Mammography screening rates	Medical record review	Significant increase in the mammography screening rate in intervention 18% to 40% versus control 18% to 18% (p<0.01).	significant improvement	not available
	Pap test screening rates	Medical record review	Significant increase in the pap test screening rate in the intervention 17% to 57% versus the control 12% to 18% (p<0.01).	significant improvement	not available

Study	Outcome	How Measured	Intervention Effect	Summary	
				Between-Group Summary	Within-Group Summary
Manfredi, 1998	Rates of pap smears among eligible patients of HMO/not of HMO	Medical record review	Rates increase in HMO patients only from 55.7% at baseline to 59.7% post-intervention but decrease in control group from 56.1% to 48.2% (not statistically significant).	significant improvement	not available
	Rates of clinical breast exams (CBE) among eligible patients of HMO/not of HMO	Medical record review	No effect of intervention in HMO patients/positive effect of intervention among non-HMO patients with rates increasing from 26.6 to 36.5% in intervention clinics and decreasing in control clinics ($p<0.05$).	significant improvement	not available
	Rates of fecal occult blood testing (FOBT) among eligible patients of HMO/not of HMO	Medical record review	Rates increased in both groups of patients. For HMO patients, rates increased from 3.2% to 12.5% in the intervention clinics with decrease in control clinics ($p<0.05$). In non-HMO patients, rates increased from 4.5% to 5.2% in intervention clinics while de	significant improvement	not available
	Rates of mammograms among eligible patients of HMO/not of HMO	Medical record review	No effect of intervention in either group of patients.	no improvement	not available
McCarthy, 1997	Mammography screening rates	Computerized audit of billing records	Prior to the intervention, the proportion of visits in which women were up-to-date was 68% in the intervention clinic and 66% in each control clinic. At the end of evaluation, there was an absolute increase of 9% in the intervention group (95% CI 2-16%) while one control clinic had an absolute increase of 1% (95%. CI:-5, 7%) and the other has an absolute decrease of 2% (95%. CI: - 3, 5%).	not available	significant improvement
**Adult ,Tobacco Cessation**					
Ahluwalia, 1999	Smoking cessation counseling by provider; ASK if patient smokes cigarettes or any other form of tobacco	Patient report	Odds ratio of "ASK" between intervention and control: if smokes cigarettes 3.97 (2.87, 5.49); if uses other tobacco products 1.40 (0.91, 2.18).	significant improvement	not available
	Smoking cessation counseling by provider; ASSIST how or when to quit smoking	Patient report	Odds ratio of "ASSIST" between intervention and control groups: how to quit 1.60 (0.78, 3.31); set a date 0.74 (0.16, 3.34).	improvement	not available
	Smoking cessation counseling by provider; ADVISE to quit smoking	Patient report	Odds ratio of "ADVISE" between intervention and control groups 1.75 (1.29, 2.35).	significant improvement	not available
	Smoking cessation counseling by provider; ARRANGE follow-up or refer to specialist	Patient report	Odds ratio of "ARRANGE" between intervention and control 1.97 (1.20, 3.24).	significant improvement	not available

Study	Outcome	How Measured	Intervention Effect	Summary	
				Between-Group Summary	Within-Group Summary
Allen, 1998	Protocol adherence	Patient report	Only 16% of the control group patients compared to nearly 70% of the intervention patients reported that their physician urged them to quit smoking.	improvement	not available
	3 month quit rate	Patient report	6.1% for intervention group versus 5.2% for control group (p=not specified).	no improvement	not available
	12 month quit rate	Saliva cotinine tests	3.2% for intervention group versus 2.8% for control group, (p=not specified).	no improvement	not available
	12 month quit rate	Patient report	8.4% for intervention group versus 10.6% for control group (not significant).	no improvement	not available
	3 month quit rate	Saliva cotinine tests	2% for intervention group versus 1.8% for control group (not significant).	no improvement	not available
**Adult, Cholesterol**					
Keyserling, 1997	Difference in total cholesterol among participants not on lipid lowering therapy at one year	Blood tests	Average reduction of 0.14 mmol/L (5.3 mg/dL) greater in intervention group (p=0.01).	significant improvement	not available
	Clinician dietary counseling behavior	Medical record review	Cholesterol mentioned in progress note for 10% of intervention patients and 30% of control patients on first routine follow-up (statistical significance not assessed).	no improvement	not available
	Referral for dietary counseling	Medical record review	9% of control group patients compared to 2% of intervention patients referred for dietary counseling (statistical significance not assessed).	no improvement	not available
	Number patients taking lipid lowering medication	Medical record review	8% of intervention patients compared to 15% of control group patients were taking lipid-lowering medication on follow-up (p=0.05).	no improvement	not available
	Difference in total cholesterol among all study participants at one year	Blood tests	Averaged over 1 year follow-up, the total cholesterol reduction in the intervention group was 0.08 mmol/L (3.0 mg/dL) greater than in control group (p=0.09).	improvement	not available
	Dietary habits	Patient report, dietary risk assessment	Sustained decrease in both groups with average reduction in the intervention group significantly more than for the control group (p>0.001).	significant improvement	not available

Study	Outcome	How Measured	Intervention Effect	Summary	
				Between-Group Summary	Within-Group Summary
	Difference in LDL among all study participants at one year	Blood tests	Average reduction 0.10 mmol/L (3.7 mg/dL) greater in intervention group (p=0.08).	improvement	not available
**PREVENTION, CHILDREN**					
**Health Behavior Screening**					
Schubiner, 1994	Total time of interview	Videotaped encounters	Physicians in intervention group had shorter visits than physicians in the control group.	improvement	not available
	Accuracy of physician assessment of patient need for interventions in each "Safe Times" topic area	Compared to "gold standard" of psychologist interview	Physicians in the intervention group had higher agreement with psychologist ratings in identification of subjects at risk for depression and family conflicts than the physicians in the control group.	improvement	not available
	Patient satisfaction	Medical Interview Satisfaction Scale	No difference in satisfaction between groups.	no improvement	not available
	Time spent making assessment versus counseling in each area	Videotaped encounters	Physicians in intervention group spent less time in assessment rather than counseling in each of the "Safe Times" areas (p<0.05).	significant improvement	not available
	Total time spent in each of the "Safe Times" topics	Videotaped encounters	Physicians in intervention group spent less time discussing each of the specific "Safe Times" topics.	improvement	not available
	Time spent discussing "Safe Times" topics	Videotaped encounters	Physicians in intervention group spent less time discussing all "Safe Times" topics.	improvement	not available
**Injury Prevention in Children**					
Gielen, 2001	Safety practices	Self report and home-observation	Groups did not differ.no improvement	not available	
	Parental knowledge and beliefs regarding injury prevention	Follow-up interview	Groups did not differ.	no improvement	not available
	Satisfaction	Patient report	Patients in the intervention group rated the help they received with safety topics significantly higher than patients in the control group (p=0.01).	significant improvement	not available
	Injury prevention counseling	Audiotape analysis	Mention of safety strategies was higher in intervention group (9.4 +/- 6.8) than control group (3.7 +/- 3.2).	improvement	not available

Study	Outcome	How Measured	Intervention Effect	Summary	
				Between-Group Summary	Within-Group Summary
**Well Baby Care**					
Hornberger, 1996	Adequacy of interpretation	Audiotape analysis	The remote simultaneous translation group had a 13% lower rate of inaccurately interpreted mother utterance per visit than the proximate consecutive service (most were omissions).	improvement	not available
	Number of questions asked by mother	Audiotape analysis	Significantly more questions were asked by mothers in the remote simultaneous translation than with the proximate consecutive translation service (no p-values given).	improvement	not available
	Provider preference for type of intervention	Provider self report	Providers preferred remote simultaneous translation to proximate consecutive translation.	improvement	not available
	Duration of encounter	Audiotape analysis	No significant difference between intervention and control.	no improvement	not available
	Number of explanations by mother	Audiotape analysis	The number of mother explanations per visit was 22% more in the remote simultaneous translation group than in the proximate consecutive translation group visits.	improvement	not available
	Number of explanations by physicians	Audiotape analysis	The number of physician explanations per visit was 18% more in the remote simultaneous translation group than in the proximate consecutive translation group visits.	improvement	not available
	Patient preference for type of intervention	Patient self report	All 17 mothers who experienced both types of translation and who responded to the survey preferred remote simultaneous translation.	improvement	not available
	Patient-physician eye-to-eye contact	Provider self-report	Physician reported that eye-to-eye contact was improved in remote simultaneous translation group.	improvement	not available
**MENTAL HEALTH**					
**Alcohol**					
Burge, 1997	Addiction Severity Index (ASI) Family Score	Interview; medical record review	Decreased over time in all groups (p=0.000), but decrease more significant for physician intervention group at 12 months (p=0.003).	significant improvement	significant improvement
	Addiction Severity Index (ASI) Employment Score	Interview; medical record review	No change over time in any group (not significant) at 12 months.	no improvement	no improvement
	Addiction Severity Index (ASI) Alcohol score	Interview; medical record review	Decreased over time in all groups (p=0.000). No difference between groups at 12 months.	no improvement	significant improvement
	Drinks per week	Interview; medical record review	Decreased over time for all groups (p=0.000). No difference between groups at 12 months.	no improvement	significant improvement
	Addiction Severity Index (ASI) Medical Score	Interview; medical record review	Decreased over time in all groups (p=0.000) at 12 months.	no improvement	significant improvement
	Mean Corpuscular Volume (MCV)	Blood test	Decreased over time in all groups (p=0.000). No difference between groups at 12 months.	no improvement	significant improvement

Study	Outcome	How Measured	Intervention Effect	Summary	
				Between-Group Summary	Within-Group Summary
	Liver enzymes (SGot, SGPt, GGT)	Blood test	No difference between groups at 12 months. SGoT decreased over time (p=0.000) in all groups.	no improvement	significant improvement
**Depression**					
Callahan, 1994	Percentage of patients with depression diagnosis recorded at 6 months	Medical record review	32.3% (intervention) versus 12.1% (control) (p=0.002).	significant improvement	not available
	Percentage of patients with newly-prescribed antidepressant at 6 months	Medical record review	26.0% (intervention) versus 8.0% (control) (p=0.01).	significant improvement	not available
	Percentage of patients referred to psychiatry at 6 months	Medical record review	13.7% (intervention) versus 12.1% (control) (no significant differences).	no improvement	not available
	Depression	Hamilton Depression Scale	Both groups showed statistically significant improvement in depression at 6 months (no significant difference between groups).	no improvement	not available
	Percentage of patients in whom drug associated with depression was discontinued	Medical record review	22.0% (intervention) versus 23.0% (control) (no significant difference).	no improvement	not available
Miranda, 2003	Depression	Composite International Diagnostic Interview (CIDI)	At 6 and 12 months, patients in both intervention groups were less likely than patients in control group to meet criteria for probable depressive disorder (39.9% versus 49.9%, p=0.001). In subgroup analysis only African American and Latino patients were significantly less likely to screen positive for depression, whereas the difference was not significant for white patients.	significant improvement	not available
	Appropriate depression care	Not specified	At 6 and 12 months, all patients (African American, Latino, and white) in both intervention groups were more likely than controls to receive counseling or to receive anti-depressant medications (50.9% versus 39.7%, p<0.001).	significant improvement	not available
	Employment	Telephone and mailed survey	At 6 and 12 months, there were no improvements for African American or Latino patients in employment rates, however, there were improvements for white patients.	no improvement	not available

Study	Outcome	How Measured	Intervention Effect	Summary	
				Between-Group Summary	Within-Group Summary
**OTHER CLINICAL AREAS**					
**Acute Respiratory Infections**					
Harris, 2003	Antibiotic prescription rates for acute bronchitis	Medical record review	The proportion of patients with acute bronchitis decreased from 58% in the baseline period to 30% and 24% among patients exposed to the limited and full interventions (p<0.001 for intervention groups versus baseline). No differences between groups.	no improvement	significant improvement
	Antibiotic prescription rates for non-specific upper respiratory tract infections	Medical record review	Antibiotic prescriptions for nonspecific upper respiratory tract infections decreased from 14% to 3% and 1% in the limited and full intervention groups (p<0.001 for intervention groups versus baseline). No difference between limited and full intervention groups.	no improvement	significant improvement
**Asthma**					
Evans, 1997	Patient education by physician and nurse	Patient report	Patient reported education by physicians was 71% for intervention versus 58% for control patients (p<0.01), and by nurses was 61% for intervention versus 41% for control patients (p<0.05).	significant improvement	not available
	Continuity of care	Average number of visits made by children with asthma per year	Average number of visits made by children with asthma increased in intervention clinics from baseline through both follow-up periods (2.15 in 1992 versus 2.42 in 1993 versus 1.41 in 1991, p<0.001) and remained the same in control clinics.	not available	significant improvement
	Use of appropriate medicines	Proportion of patients with asthma who had each type of medication dispensed	In the intervention clinics, a higher proportion of patients with asthma compared to controls were given inhaled anti-inflammatory medication (17 versus 3, p<0.001), spacers devices (28 versus 3, p<0.001), and anti-inflammatory meds (68 versus 50, p<0.05).	significant improvement	not available
	Continuity of care	Proportion of patients treated for asthma in 1 year who returned for treatment the next year	Percent of patients with asthma in year 1 who returned in year 2 increased in intervention clinics 16% to 32% (p<.0002) and decreased slightly in control clinics 14% to 12% (not significant).	not available	significant improvement
	Identification/ treatment of asthma patients	Number of asthmatic patients per 1000 clinic patients	Intervention clinics doubled (p<.001) rate at which they identified asthma patients with no change in control clinics during both 1st and 2nd follow-up years.	not available	significant improvement
**Chronic Renal Disease**					
Harris, 1998	All outpatient clinic visits (years 3-5 after enrollment)	Medical record review	Intervention group patients had significantly more outpatient clinic visits overall than did control group patients, 26 versus 18 (p<0.001).	significant improvement	not available
	Blood pressure (years 3-5 after enrollment)	Medical record review	No significant differences in systolic and diastolic blood pressures between groups.	no improvement	not available

Study	Outcome	How Measured	Intervention Effect	Summary: Between-Group Summary	Summary: Within-Group Summary
	Emergency department visits (years 3-5 after enrollment)	Medical record review	No significant difference in emergency department visits between groups.	no improvement	not available
	Hospitalizations (years 3-5 after enrollment)	Medical record review	No significant difference in number of hospitalizations between groups.	no improvement	not available
	Calcium channel blocker use (years 3-5 after enrollment)	Medical record review	Patients in the intervention group were more likely to be on a calcium channel blocker than were patients in control group, 49% versus 35% (p=0.003).	no improvement	not available
	Ophthalmology clinic visits (years 3-5 after enrollment)	Medical record review	Patients in the intervention group had a greater number of ophthalmology appointments than did patients in the control group, 1.8 versus 1.2 (p=0.07).	improvement	not available
	Cumulative 5 year mortality	Death certificate from Indiana State Department of Health, hospital death summaries, discharge status reports, autopsy reports	No significant difference in intervention (29%) versus control (33%) group in cumulative 5 year mortality.	no improvement	not available
	Change in renal function (years 3-5 after enrollment)	Medical record review	Intervention group patients had lower creatinine clearance than did control group patients, 30 versus 34 (p=0.10).	no improvement	not available
**Emergency Systems**					
Kellermann, 1993	Neurological deficits	Physician/nurse report	Neurological outcomes for survivors of both groups were similar.	no improvement	not available
	Survival to hospital discharge	Hospital report	Patients treated by automated external defibrillator equipped responders were no more likely to survive to hospital discharge.	no improvement	not available
	Survival to hospital admission	Written report	Patients treated by automated external defibrillator equipped responders were no more likely to survive to hospital admission.	no improvement	not available

Study	Outcome	How Measured	Intervention Effect	Summary	
				Between-Group Summary	Within-Group Summary
**End of Life**					
Dexter, 1998	Completion of either Instruction or Proxy Directive	Patients complete either Directive	The percentage of patients who completed either directive was 15% in the instruction and proxy group, 7% in the instruction group, 3% in the proxy group, and 4% in the control group.	improvement	not available
	Discussion of advance directives	Patient report	Compared with control, dissemination of advanced directives had OR of 7.7 (3.4 to 18) in instruction and proxy group; 4.4 (2.1 to 9.4) in instruction group; and 2.5 (1.1 to 5.5) in proxy group.	significant improvement	not available
	Initiation of discussion	Patient report	Discussions initiated by physician in 86% of instances in instruction and proxy group, 77% in instruction group, 43% in proxy group, and 38% in control group.	improvement	not available
	Completion of Proxy Directive	Completion of form by patient	The percentage of patients who complete a Proxy Directive was 8% in the instruction and proxy group, 4% in the instruction group, 1% in the proxy group, and 2% in the control group.	improvement	not available
	Completed Instruction Directive	Completion of form by patient	The percentage of patients who completed an instruction directive in the instruction and proxy group was 15%, 7% in instruction group, 2% in proxy group, and 2% in control group.	improvement	not available

[a] The overall summary columns are presented for both comparisons between intervention arms and comparisons within intervention arms (such as when there was a pre- and a post-intervention assessment), as some studies presented one but not the other, whereas other studies presented both comparisons. For this purpose, “significant improvement” refers to outcomes that showed an effect that was statistically significantly better, “improvement” refers to outcomes that showed a better effect that was not statistically significant, “no improvement” refers to outcomes that were not improved, “negative effect” refers to outcomes that were worse after the intervention, and “not available” refers to outcomes for which no comparison was made.
